# Can PD-L1 expression evaluated by biopsy sample accurately reflect its expression in the whole tumour in gastric cancer?

**DOI:** 10.1038/s41416-019-0515-5

**Published:** 2019-07-09

**Authors:** Kohei Yamashita, Masaaki Iwatsuki, Kazuto Harada, Yuki Koga, Yuki Kiyozumi, Kojiro Eto, Yukiharu Hiyoshi, Takatsugu Ishimoto, Shiro Iwagami, Yoshifumi Baba, Yuji Miyamoto, Naoya Yoshida, Yoshihiro Komohara, Jaffer A. Ajani, Hideo Baba

**Affiliations:** 1 Department of Gastroenterological Surgery, Graduate School of Medical Sciences, Kumamoto University, Kumamoto, Japan; 2 Department of Gastrointestinal Medical Oncology, The University of Texas MD Anderson Cancer Center, Houston, TX 77030 USA; 3 Department of Cell Pathology, Graduate School of Medical Sciences, Kumamoto University, Kumamoto, Japan

**Keywords:** Gastric cancer, Tumour heterogeneity

## Abstract

Programmed death ligand 1 (PD-L1) expression as a predictive biomarker for programmed cell death 1 (PD-1) inhibitor efficacy in gastric cancer (GC) remains controversial. We hypothesised that the conflicting results may be due to the inaccurate assessment of PD-L1 expression using biopsy samples. A total of 191 patients with GC who received radical resection were enrolled. PD-L1 expressions in biopsy and paired resected samples by immunohistochemistry staining were compared according to the number of biopsies. The numbers of PD-L1-positive patients determined by biopsy and resected samples were 89 (46.6%) and 135 (70.1%), respectively. The accordance rate was 64.4% (κ = 0.31). Single biopsy showed a lower accordance rate compared with multiple biopsies. Our study revealed that single biopsy cannot fully reflect PD-L1 expression in the whole tumour in GC. Multiple biopsies are recommended for accurate diagnosis of PD-L1 expression in GC.

## Background

Immune therapy targeting programmed cell death 1 (PD-1) and its ligand programmed death ligand 1 (PD-L1) demonstrated favourable therapeutic effects in gastric cancer (GC) in several clinical trials.^1,2^ PD-L1 expression has been considered a potential biomarker for treatment efficacy in several types of cancer, including melanoma and non-small-cell lung carcinoma (NSCLC).^3^ However, whether PD-L1 expression is a predictive biomarker for PD-1/PD-L1 inhibitor efficacy in GC remains controversial.^1,4^

GC shows a strong histological heterogeneity in primary lesions, as various histological and differentiation types are frequently observed in the same samples. Intratumoural heterogeneity is often an obstacle for accurate assessment of tumour profiles and determining treatment strategy.^5^ We hypothesised that PD-L1 expression evaluated by biopsy samples might differ from PD-L1 expression in the whole tumour because of intratumoural heterogeneity. To confirm whether PD-L1 expression evaluated by biopsy specimen accurately reflects its expression in the whole tumour, we compared PD-L1 expression of biopsy samples with those of matched resected samples and examined the correlations between the number of biopsies and accurate diagnosis of PD-L1 expression.

## Methods

### Patients and samples

A total of 191 patients with advanced GC with tumour depth below the muscularis propria layer were included (Supplemental Table 1). The patients received radical gastrectomy without pretreatment between 2005 and 2014 in Kumamoto University Hospital. Paired biopsy and resected samples from the same patients were used for PD-L1 immunohistochemistry. The study procedures were approved by the institutional review board (No. 1037), which waived the requirement for informed consent because of the retrospective nature of the study.

### Immunohistochemistry

Formalin-fixed paraffin-embedded GC tissue samples were sectioned (5 μm) and deparaffinised. Heat-induced antigen retrieval was performed in antigen retrieval solution (pH 9, Histofine; Nichirei Biosciences, Tokyo, Japan) with a steamer autoclave at 121 °C for 15 min. Endogenous peroxidase activity was blocked using 3% hydrogen peroxide, and slides were incubated with rabbit monoclonal primary antibody against PD-L1 (1:200 dilution; clone E1L3N, 13684S; Cell Signaling Technology, Danvers, MA, USA) overnight at 4 °C. Slides were incubated with a secondary antibody (anti-rabbit EnVision™+/horseradish peroxidase; Dako Japan, Tokyo, Japan) and counterstained with haematoxylin (Supplementary Fig. 1).

### Evaluation of PD-L1 expression

PD-L1 expressions in biopsy and resected samples were assessed by one of the investigators (K.Y.) and an experienced pathologist (Y.K.) who were unaware of the clinical data. The observers individually calculated the combined positive score (number of PD-L1-positive cells [tumour cells, macrophages and lymphocytes] divided by the total number of tumour cells, multiplied by 100) and averaged scores to establish consensus. PD-L1 positivity was defined when the average score was 1 or greater, as described previously.^2^ All samples included at least 100 viable tumour cells, which is the recommended tumour volume for scoring.^6^

### Statistical analysis

To examine the consistency of PD-L1 expression between biopsy and resected samples, the kappa coefficient was calculated. The accordance rate was compared using Chi-square tests based on the number of biopsies. Statistical analyses were performed by JMP^®^ version 13.1 software (SAS Institute). *p* < 0.05 was considered statistically significant.

## Results

### PD-L1 expression in biopsy and resected samples

Among the 191 patients, 89 (46.6%) patients showed PD-L1-positive biopsy and 135 (70.1%) patients showed PD-L1-positive resected samples (Table 1). The accordance rate of PD-L1 positivity between the biopsy and resected samples was 64.4% and the kappa coefficient value was equal to 0.31 (poor agreement). False negativity (negative PD-L1 in biopsy and positive PD-L1 in a resected specimen) was observed in 57 cases (29.8%) and was the main factor for the high discordance of PD-L1 expression between the biopsy and resected specimen.

**Table 1:** Comparison of PD-L1 positivity between biopsy and resected specimens

	Resected specimen		
	Positive (≧1%)	Negative (<1%)	Total
Biopsy specimen			
Positive (≧1%)	78	11	89
Negative (<1%)	57	45	102
Total	135	56	191
Accordance rate (%)		64.4	
Kappa coefficient (value)		0.31	

### Correlation between the number of biopsies and accordance rate

**Fig. 1:**
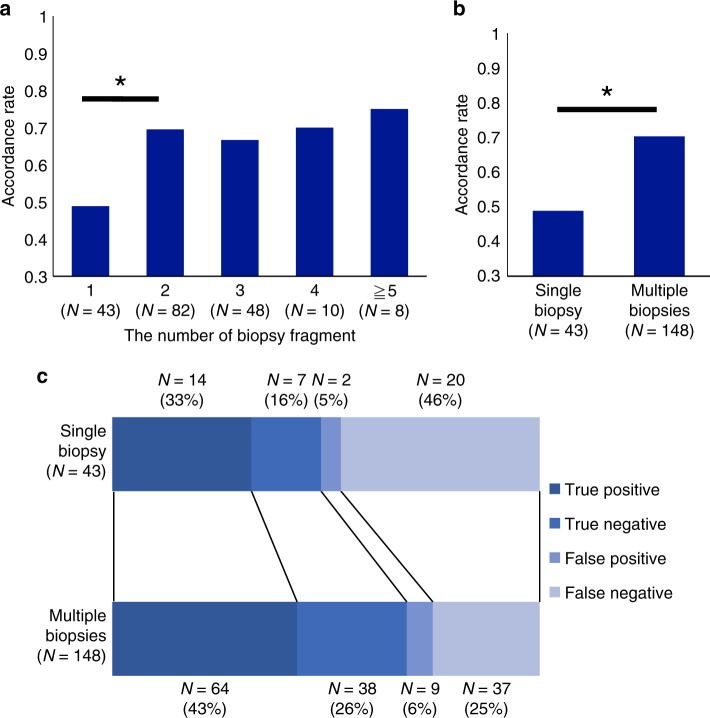
Correlation between the number of biopsies and accordance rate. **a** Comparison of the accordance rate of PD-L1 expression between biopsy and resected samples according to the number of biopsies. *p *<* 0.05. **b** Comparison of the accordance rate of PD-L1 expression between biopsy and resected samples according to single or multiple biopsies. *p < 0.05. **c** Distribution of PD-L1 expression evaluated by biopsy according to single or multiple biopsies

To assess the impact of the number of biopsies on the accuracy of PD-L1 evaluation, the correlation between the number of biopsies and accordance rate of PD-L1 expression was examined (Fig. 1). Among the 191 cases, single biopsy was performed in 43 cases (22.5%) and multiple biopsies were performed in 148 cases (77.5%). The accordance rate of single-biopsy cases was significantly lower (48.8%) than that of multiple biopsy cases (68.9%) (*p* < 0.05, Fig. 1a, b). Moreover, single-biopsy cases showed low positive and negative percent agreement (Supplementary Table 2). Multivariate logistic regression analysis demonstrated that single biopsy was associated with discordance (Supplementary Table 3). False-negative cases comprised 46% of single-biopsy cases, whereas the proportion of true-positive cases and true-negative cases increased by 10% in multiple biopsy cases (Fig. 1c).

## Discussion

Our results showed that biopsy could not fully reflect PD-L1 expression of the whole tumour in GC. PD-L1 positivity in biopsy samples was observed in 46.6% of the GC patients, which was consistent with previous reports.^2^ In contrast, PD-L1 positivity was observed in 70.7% of paired resected samples. Moreover, the accordance rate of PD-L1 expression between biopsy and resected samples was 64.4%, with a marked amount of false-negative cases. Notably, a significantly lower accordance rate was observed among GC patients with single biopsy than those with multiple biopsies. Therefore, multiple biopsies are recommended to improve the accurate diagnosis of PD-L1 expression in GC.

The discordance of PD-L1 expression between biopsy and resected samples was also reported in NSCLC.^7^ However, the heterogeneity of PD-L1 expression specific to GC may be an important contributing factor for the discordance. Previous studies reported GC cases in which PD-L1-expressing cells localised only in the tumour centre.^8^ Therefore, we hypothesised that biopsy samples might not fully reflect the PD-L1 expression in the whole tumour in GC, because biopsy samples are usually obtained from the mucosal part of the tumour. Notably, we demonstrated that single biopsy is insufficient to overcome the tumour heterogeneity for the accurate assessment of PD-L1 expression. In evaluating HER2 expression in GC, several guidelines have indicated that a minimum of five biopsy specimens should be obtained to account for intratumoural heterogeneity and to provide sufficient tumour specimens for diagnosis and biomarker testing.^9^ We also found a higher accordance rate in cases with more than five biopsies (Supplementary Table 2). Further studies using more samples, including more than five biopsies, are needed to determine the optimal number of biopsies for PD-L1 evaluation.

This study has several limitations. First, patients who underwent pretreatment for GC were excluded. Previous clinical trials evaluated PD-L1 expression in patients with treatment history. However, PD-L1 expression can be dynamically modulated in response to treatment.^10^ Therefore, we excluded patients who underwent pretreatments to avoid this alteration bias. Second, we did not assess whether the discordance of PD-L1 evaluation between the biopsy and resected samples affects the prediction of the PD-1 inhibitor efficacy. Further studies are needed to confirm the significance of PD-L1 expression in the whole tumour as a predictive biomarker for PD-1 inhibitors.

In conclusion, we demonstrate that biopsy cannot fully reflect the PD-L1 expression of the whole tumour in GC. Multiple biopsies are recommended to improve accurate diagnosis of PD-L1 expression. This study is clinically meaningful in discussing the significance of PD-L1 expression as a predictive biomarker for PD-1/PD-L1 inhibitors in GC.

## Supplementary information

Supplementary FigureS1

Supplementary TableS1

Supplementary TableS2

Supplementary TableS3

## Data Availability

Individual-level data may be shared on request if all legal and ethical requirements are met. Requests should be sent to the corresponding author.
