# Supplementary material for: Can PD-L1 expression evaluated by biopsy sample accurately reflect its expression in the whole tumour in gastric cancer?
Source: Br J Cancer. 2019 Jul 9;121(3):278–80. doi: 10.1038/s41416-019-0515-5 (PMC6738080; doi:10.1038/s41416-019-0515-5)
Supplement: Supplementary file 1 — Supplementary FigureS1 [file 41416_2019_515_MOESM1_ESM.pptx]

## Slide 1
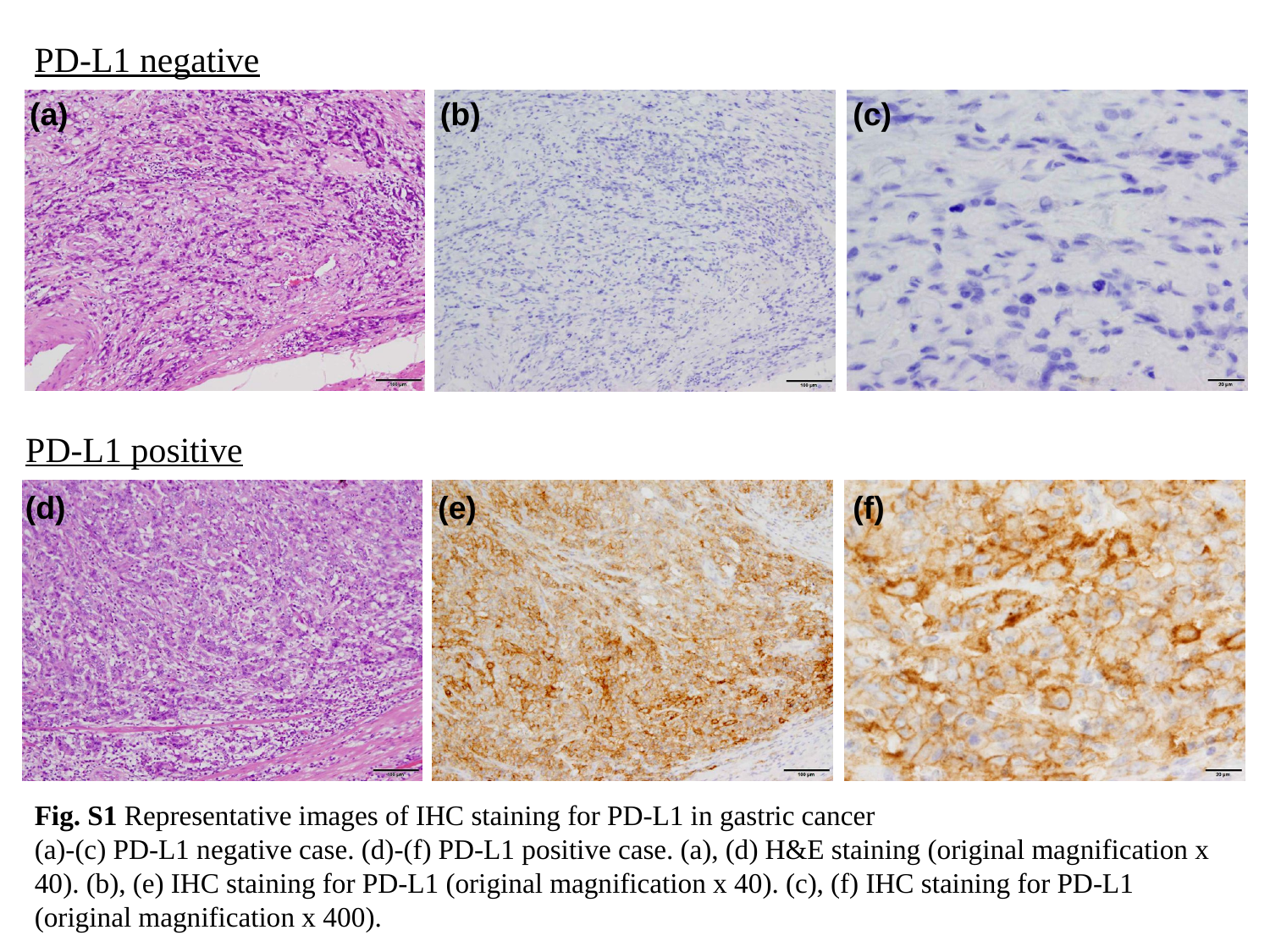

PD-L1 negative
(a)
(b)
(c)
PD-L1 positive
(d)
(e)
(f)
Fig. S1 Representative images of IHC staining for PD-L1 in gastric cancer
(a)-(c) PD-L1 negative case. (d)-(f) PD-L1 positive case. (a), (d) H&E staining (original magnification x 40). (b), (e) IHC staining for PD-L1 (original magnification x 40). (c), (f) IHC staining for PD-L1 (original magnification x 400).
