# Supplementary material for: Can PD-L1 expression evaluated by biopsy sample accurately reflect its expression in the whole tumour in gastric cancer?
Source: Br J Cancer. 2019 Jul 9;121(3):278–80. doi: 10.1038/s41416-019-0515-5 (PMC6738080; doi:10.1038/s41416-019-0515-5)
Supplement: Supplementary file 2 — Supplementary TableS1 [file 41416_2019_515_MOESM2_ESM.doc]

| **Table S1**. Patient characteristics | |
| --- | --- |
|  | Total  (N = 191) |
| Age (years) | 72 (22 - 94) |
| Sex  Male  Female | 135 (70.7 %)  56 (29.3 %) |
| Tumor size (mm) | 50 (10 – 200) |
| Tumor location  Upper  Middle  Lower | 66 (34.6 %)  68 (35.6 %)  57 (29.8 %) |
| Histological type  Differentiated  Undifferentiated | 96 (50.3 %)  95 (49.7 %) |
| T stage  T2  T3  T4 | 50 (26.2 %)  90 (47.1 %)  51 (26.7 %) |
| N stage  N0  N1  N2  N3 | 96 (50.3 %)  33 (17.3 %)  31 (16.2 %)  31 (16.2 %) |
| Stage  I  II  III | 37 (19.4 %)  85 (44.5 %)  69 (36.1 %) |
| Data are shown as number of cases (%) or mean number (range). | |
