# Supplementary material for: Can PD-L1 expression evaluated by biopsy sample accurately reflect its expression in the whole tumour in gastric cancer?
Source: Br J Cancer. 2019 Jul 9;121(3):278–80. doi: 10.1038/s41416-019-0515-5 (PMC6738080; doi:10.1038/s41416-019-0515-5)
Supplement: Supplementary file 3 — Supplementary TableS2 [file 41416_2019_515_MOESM3_ESM.doc]

| **Table S2.** Relationships between the number of biopsies and accordance rate | | | | |
| --- | --- | --- | --- | --- |
| The number of biopsies | PPA (%) | NPA (%) | OAR (%) | Κ coefficient |
| 1 (N=43) | 41.2 | 77.8 | 48.8 | 0.11 |
| 2 (N=82) | 65.5 | 79.2 | 69.5 | 0.38 |
| 3 (N=48) | 58.6 | 79.0 | 66.7 | 0.35 |
| 4 (N=10) | 57.1 | 100 | 70.0 | 0.44 |
| ≧5 (N=8) | 71.4 | 100 | 75.0 | 0.38 |
| < 5 (N=183) | 57.0 | 80.0 | 63.9 | 0.30 |
| ≧5 (N=8) | 71.4 | 100 | 75.0 | 0.38 |
| Total (N=191) | 57.8 | 80.4 | 64.4 | 0.31 |

PPA Positive percent agreement, NPA Negative percent agreement, OAR Overall accordance rate
