# Supplementary material for: Can PD-L1 expression evaluated by biopsy sample accurately reflect its expression in the whole tumour in gastric cancer?
Source: Br J Cancer. 2019 Jul 9;121(3):278–80. doi: 10.1038/s41416-019-0515-5 (PMC6738080; doi:10.1038/s41416-019-0515-5)
Supplement: Supplementary file 4 — Supplementary TableS3 [file 41416_2019_515_MOESM4_ESM.docx]

| **Table S3.**  Univariate and multivariate logistic regression analysis for discordance of PD-L1 expression between biopsy and resected specimen | | | | | | | |
| --- | --- | --- | --- | --- | --- | --- | --- |
|  | Univariate analysis | | |  | Multivariate analysis | | |
|  | OR | 95% CI | *p*-value |  | OR | 95% CI | *p*-value |
| Age ≧ 65 years | 1.19 | 0.61 – 2.34 | 0.61 |  |  |  | NS |
| Male | 0.80 | 0.42 – 1.52 | 0.50 |  |  |  | NS |
| Upper location | 0.77 | 0.40 – 1.50 | 0.45 |  |  |  | NS |
| Tumor diameter ≧ 50mm | 0.62 | 0.34 – 1.13 | 0.12 |  |  |  | NS |
| Undifferentiated type | 1.02 | 0.56 – 1.84 | 0.96 |  |  |  | NS |
| Serosa invasion | 1.55 | 0.80 – 2.99 | 0.19 |  |  |  | NS |
| Lymph node metastasis | 1.22 | 0.67 – 2.21 | 0.51 |  |  |  | NS |
| Single biopsy | 2.32 | 1.16 – 4.64 | 0.02 |  | 2.49 | 1.20 – 5.17 | 0.01 |
| OR Odds ratio, CI Confidence interval | | | | | | | |
